# A mapping exercise to identify the strengths, and gaps in knowledge translation activities at Cochrane South Africa

**DOI:** 10.11604/pamj.2023.45.64.38075

**Published:** 2023-05-29

**Authors:** Anelisa Jaca, Chanelle Mulopo, Charles Shey Wiysonge, Bey-Marrié Schmidt

**Affiliations:** 1 Cochrane South Africa, South African Medical Research Council, Cape Town, South Africa,; 2 School of Public Health, University of the Western Cape, Bellville, South Africa,; 3 HIV and other Infectious Diseases Research Unit, South African Medical Research Council, Durban, South Africa

**Keywords:** Knowledge translation, research evidence, dissemination, practice

## Abstract

Knowledge translation (KT) is a set of activities or processes for synthesising, disseminating, and applying research evidence in decision-making for the benefit of society. For KT to be successful, it is paramount for researchers to play an active role in encouraging evidence uptake and use in decision-making. We carried out a mapping exercise and interviews with research cluster heads at Cochrane South Africa (CSA) of the KT activities and processes being implemented (or are planned for implementation). We organized the mapping and interview results according to the KT themes described in the Cochrane KT framework. The KT framework comprises six themes, namely, (i) prioritization and co-production of research evidence; (ii) building a sustainable infrastructure for knowledge translation; (iii) engaging with audiences for knowledge exchange or dialogue; (iv) packaging, communication and dissemination which entails disseminating research to users; (v) building audience capacity to use evidence or training activities; and (vi) advocacy or improving the culture of using evidence. Through the mapping exercise and interviews, we learned that CSA researchers excelled in implementing activities and processes linked to most of the KT themes, including producing different types of systematic reviews and providing reliable evidence for health decision-making. Cochrane South Africa (CSA) researchers are also involved in mentoring and training postgraduate students and various health decision-makers (e.g., health professionals, guideline panels and policy-makers). While they excel in the above-mentioned activities, “packaging, communication, and dissemination of research evidence” (theme iv) was identified as an area of improvement.

## Commentary

Achieving Universal Health Coverage (UHC) is a priority in many low- and middle-income countries and has resulted in an increased demand for research evidence to inform health policy and practice decision-making [1-3]. Researchers play an important role in promoting the uptake and use of the best available research evidence in health policy and practice decision-making [4,5]. However, there are various challenges that may hinder the extent to which research is translated into policy and practice. Challenges may relate to researchers and decision-makers individual (skills and motivations), institutional (resources and buy-in) and contextual (political agenda) factors [6]. To overcome these challenges, we recommend an Integrated Knowledge Translation (IKT) approach to foster mutually beneficial research and engagement between researchers and decision-makers. Integrated Knowledge Translation (IKT) can facilitate research co-production, knowledge exchange, dialogue and capacity strengthening between various health decision-makers (e.g. researchers and policy-makers), with the goal of improving healthcare delivery, system performance and ultimately patient outcomes [7]. For effective IKT, researchers engage relevant health decision-makers from the start of the research process and simplify and package research results using different tools and mechanisms suitable for different audiences [8]. Examples of different tools and mechanisms for translating research results to various health decision-makers are scientific articles, presentations, webinars, social media, press releases, policy briefs, and infographics.

Cochrane South Africa (CSA) is a non-profit organization that produces systematic reviews (and other types of research) on high priority health topics and applies an IKT approach to support the uptake and use of research evidence in policy and practice decision-making [9]. Cochrane South Africa´s IKT work is guided by the Cochrane Knowledge Translation (KT) framework [10]. The framework describes six themes on the types of KT activities and processes to be implemented within Cochrane globally. We used the framework to guide a mapping exercise conducted in July 2022 to: (a) identify and classify the IKT activities and processes implemented by researchers at CSA; and (b) explore the barriers and facilitators of implementing an IKT approach, as a way of identifying current gaps and areas of improvement. Cochrane South Africa is organized into research clusters, as such the mapping exercise involved interviewing the four research cluster heads and asking them to complete a spreadsheet capturing the activities and processes, they are implementing or are planning to implement, as per the framework.

The research cluster heads were asked to classify their work according to the following six themes: (i) prioritization and co-production of research evidence (e.g., systematic reviews), which involves producing reviews which meet the needs of users; (ii) building a sustainable infrastructure for knowledge translation, which is about developing capacity in KT; (iii) engaging with audiences for knowledge exchange or dialogue to support their evidence-informed decision-making; (iv) packaging, communication, and dissemination, which entails disseminating research to users; (v) building audience capacity to use evidence or training activities which is about making Cochrane reviews accessible; (vi) advocacy or improving the culture of using evidence, which is about advocating for evidence-informed health decision-making.

Below, we summarize the key findings from the mapping exercise (Table 1). We found that researchers implemented or planned to implement activities related to five of the six KT themes. Cochrane South Africa researchers excelled in the production of systematic reviews of effectiveness, qualitative evidence synthesis, scoping reviews, rapids reviews and recommendations for guidelines, predominantly in response to the National Department of Health and World Health Organization. Cochrane South Africa researchers are also actively building their own capacity in KT; for example, all the research cluster heads attended an introductory training in KT or are working closely with colleagues who are experts in KT. Cochrane South Africa researchers are continuously engaging with health decision-making bodies, such as the World Health Organization Regional Office for Africa, National Essential Medicines List Committee, and National Advisory Group on Immunization amongst others, for knowledge exchange, capacity building and dialogue.

**Table 1:** a heat map of KT activities at Cochrane South Africa according to the six themes

KT activities at Cochrane South Africa						
**KT activities**	**Theme 1**	**Theme 2**	**Theme 3**	**Theme 4**	**Theme 5**	**Theme 6**
Researcher 1	4	5	5	2	4	4
Researcher 2	5	5	5	2	4	4
Researcher 3	4	5	5	3	4	4
Researcher 4	5	5	5	2	4	4

KT: knowledge translation

Cochrane South Africa researchers are involved in mentoring and training a wide range of health decision-makers across South Africa and other African countries and facilitating workshops and webinars to advance evidence-informed decision-making, for example, through the Cochrane Africa Network, SA GRADE Network and Historically Disadvantaged Institutions in South Africa. Although research cluster heads mentioned that the advocacy work needs to be strengthened, CSA researchers carry out several awareness-raising activities on systematic reviews and the principles of evidence-informed decision-making. Research cluster heads identified “packaging, communication and dissemination” as the KT theme needing most improvement. They specifically mentioned that improvements are needed for translating research evidence for the public. Although research cluster heads are somewhat involved in sharing research evidence via newsletters, webinars, journal articles, websites, social media (Twitter), blog shots and press releases, they questioned whether their KT work is making a difference amongst various audiences. As such, CSA researchers are interested in learning more about monitoring and evaluating their KT activities and processes and developing a KT evaluation plan that will be implemented to determine if new and ongoing KT efforts are making an impact on actions, policy and practice amongst various audiences.

## Competing interests

The authors declare no competing interests.

## Authors' contributions

All the authors have read and agreed to the final manuscript.

## References

[1] Tangcharoensathien V, Mills A, Palu T (2015). Accelerating health equity: the key role of universal health coverage in the Sustainable Development Goals. BMC Med.

[2] Conalogue DM, Kinn S, Mulligan JA, McNeil M (2017). International consultation on long-term global health research priorities, research capacity and research uptake in developing countries. Health Res Policy Syst.

[3] Edwards A, Zweigenthal V, Olivier J (2019). Evidence map of knowledge translation strategies, outcomes, facilitators and barriers in African health systems. Health Res Policy Syst.

[4] Uzochukwu B, Onwujekwe O, Mbachu C, Okwuosa C, Etiaba E, Nyström ME (2016). The challenge of bridging the gap between researchers and policy makers: experiences of a Health Policy Research Group in engaging policy makers to support evidence informed policy making in Nigeria. Global Health.

[5] Liang Z, Howard PF, Leggat SG, Murphy G (2012). A framework to improve evidence-informed decision-making in health service management. Australian Health Review.

[6] Young T, Shearer JC, Naude C, Kredo T, Wiysonge CS, Garner P (2018). Researcher and policymaker dialogue: the policy BUDDIES project in Western Cape Province, South Africa. BMJ Glob Health.

[7] Gagliardi AR, Berta W, Kothari A, Boyko J, Urquhart R (2016). Integrated knowledge translation (IKT) in health care: a scoping review. Implement Sci.

[8] Boswell C, Smith K (2017). Rethinking policy “impact” four models of research-policy relations. Palgrave Communications.

[9] Cochrane South Africa and South African Medical Research Council (2018). Cochrane South Africa Overview of Projects.

[10] Cochrane (2017). Cochrane Knowledge translation framework 2017.

